# First-principles study of interface doping in ferroelectric junctions

**DOI:** 10.1038/srep24209

**Published:** 2016-04-11

**Authors:** Pin-Zhi Wang, Tian-Yi Cai, Sheng Ju, Yin-Zhong Wu

**Affiliations:** 1 School of Mathematics and Physics, Suzhou University of Science and Technology, Suzhou 215009, China; 2 Department of Physics and Jiangsu Key Laboratory of Thin Films, Soochow University, Suzhou 215006, China

## Abstract

Effect of atomic monolayer insertion on the performance of ferroelectric tunneling junction is investigated in SrRuO_3_/BaTiO_3_/SrRuO_3_ heterostrucutures. Based on first-principles calculations, the atomic displacement, orbital occupancy, and ferroelectric polarization are studied. It is found that the ferroelectricity is enhanced when a (AlO_2_)^−^ monolayer is inserted between the electrode SRO and the barrier BTO, where the relatively high mobility of doped holes effectively screen ferroelectric polarization. On the other hand, for the case of (LaO)^+^ inserted layer, the doped electrons resides at the both sides of middle ferroelectric barrier, making the ferroelectricity unfavorable. Our findings provide an alternative avenue to improve the performance of ferroelectric tunneling junctions.

Ferroelectric (FE) materials have attracted significant interests due to their technological application in electronic devices, such as field-effect transistors (FET) and nonvolatile random access memories^1^,^2^,^3^. The inherent spontaneous electric polarization can be switched between two (or more) stable polarization states and thus can be used to modulate the screening charge at the interface^4^,^5^,^6^, or can be used as a memory state variable. Furthermore, due to the existence of ferroelectricity in nanometer scale which has been demonstrated in experiments and theory^7^,^8^,^9^,^10^,^11^,^12^,^13^. FE heterostructures, such as ferroelectric tunneling junction (FTJ), have become a very promising candidate for application in FTJ-based nanoscale transducers and future non-volatile memories with high storage density, high speed, and low power consumption^14^,^15^. FTJ is a FE film sandwiched between two metallic electrodes, the surface charges in the ferroelectric are not completely screened by the adjacent metals and the depolarization field in the barrier is not zero^16^. In general, the interface inevitably exists between the metal and the FE barrier in FTJs, and it will bring great influence on the ferroelectricity of the barrier and the transportation property of FTJs^17^. The formation of intrinsic dipole moments at the interface has been confirmed. For three types of heterostructures, i.e., vacuum/LaO/BTO/LaO, LaO/BTO, and SRO/LaO/BTO/LaO, it was found that the polar interfaces create an intrinsic electric field which is screened by electron charges leaking into the BTO barrier^18^. This made a FE dead layer near the interface, which is nonswitchable and thus is detrimental to ferroelectricity. Different terminal atomic structure of the FE barrier will also influence the performance of FTJs. It was proved that the Pt/BTO/Pt junction with TiO_2_-terminated layer is more conductive than the BaO-terminated one^19^. In addition, it was found that due to an build-in interface dipole, BaO/RuO_2_ interface in the SrRuO_3_/BaTiO_3_/SrRuO_3_(SRO/BTO/SRO) junction is unfavorable to the switchable FE polarization. Replacing one or two unit cells of BaTiO_3_ with SrTiO_3_ at this interface will alleviate this effect^20^,^21^. Therefore, interface engineering is a practical way to improve the performance of FE nanodevices.

Due to the atomic-layer control of the growth and atomic-scale measurement of composition and electronic structure at buried interfaces are possible, the atomic layer insertion becomes one of the effective interface engineering in multiferroic tunneling junction and FE heterostructures. It was proposed that when a Ni monolayer inserted at one interface in the epitaxial Fe/PbTiO_3_/Fe junction, large robust ME effects and good tunneling performances (TER and TMR) are obtained^22^. In the meantime, it was demonstrated that the insertion of the conducting layer LaNiO_3_ between the Bi_6_FeCoTi_3_O_18_ epitaxial film and the substrate is a powerful method in achieving high quality layered oxide thin films^23^. Also it was found that the existence of an additional FeO monolayer in the interface of Fe/BaTiO_3_/Fe multiferroelectric junction could lead to the vanishing critical thickness for ferroelectricity and the enhancement of ME coupling^24^.

LaAlO_3_ (LAO) is a polar perovskite oxide which consists of the alternative stacked positively charged (LaO)^+^ layer and negatively charged (AlO_2_)^−^ layer. As such, LAO can directly support electron (LaO termination) or hole (AlO_2_ termination) doping at the interface when it is deposited on non-polar oxide via electronic reconstruction^25^. This motivates us to explore the effects of polar interface on the ferroelectricity of barrier in FTJs. In this report, using the typical SRO/BTO/SRO junction as a prototype, the (AlO_2_)^−^ monolayer and (LaO)^+^ monolayer are inserted between the SRO electrode and BTO barrier. The change of crystal structure may have some influence on the results when the magnetic degree of freedom is considered^26^. Here, we focus on the polar distortion along z direction (both from ferroelectric BTO and the charged insertion layer (LaO)^+^ or (AlO_2_)^−^ in these perovskite oxides. Following the discussions of SRO/STO/LaO/STO/SRO junction and LAO/PTO heterostructures^27^,^28^, the variation of crystal structure away from *P*4*mm* does not affect the main results. Since our junction system is assumed to be deposited on the STO substrate, only the lattice constants in *z*-direction are optimized. We perform ab initial simulation on the junctions containing different types of atomic layer insertion at the interface. The FE properties and the electronic structures of the junctions are discussed, and the mechanism that causes the enhancement of ferroelectricity under the case of (AlO_2_)^−^ monolayer insertion is revealed.

## Computation details

**Figure 1:**
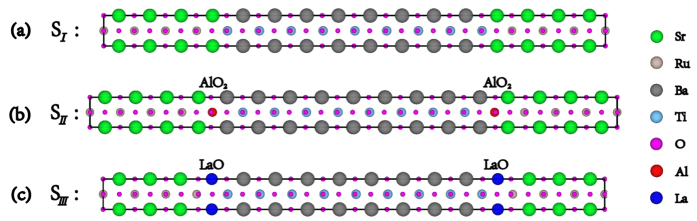
Illustration of SRO/BTO/SRO tunneling junction (a) without atomic insertion at the interface, (b) with (AlO_2_)^−^ monolayer insertion, and (c) with (LaO)^+^ monolayer insertion. Here, the thickness of BTO barrier is 8.5 unit cells, and the symbols S_*I*_, S_*II*_ and S_*III*_ are used to represent the above three structures, respectively.

To explore the influence of the inserted atomic monolayer on the FE properties of the SRO/BTO/SRO junction, we construct three types of supercells with symmetric electrodes and interfaces, namely, (SrRuO_3_)_7_-SrO-TiO_2_-(BaTiO_3_)_8_, (SrRuO_3_)_7_-SrO-AlO_2_-BaO-(TiO_2_-BaO)_8_-AlO_2_, and RuO_2_-(SrRuO_3_)_6_-LaO-TiO_2_-(BaTiO_3_)_8_-LaO, as shown in Fig. 1. One can see that the above three structures possess the same number of FE layers (8.5 L), and they correspond to the case of no interfacial insertion, (AlO_2_)^−^ atomic insertion, and LaO atomic insertion at the interface, respectively. In order to conveniently express the above supercells, the symbols S_*I*_, S_*II*_ and S_*III*_ are hereafter referred to as the FTJ structure without interface insertion, with (AlO_2_)^−^ atomic insertion, and with (LaO)^+^ atomic insertion, respectively.

First-principles calculations are performed based on density function theory (DFT) using the projector-augmented-wave (PAW) method as implemented in Vienna ab initio simulation package (VASP)^29^. The local-density approximation (LDA) for exchange and correlation is employed and the energy cutoff of 500 eV is selected for the plane-wave expansion. The ions are relaxed until the Hellmann-Fynman forces are less than 20 meV/Å, and the 8 * 8 * 1 k-points meshes are used for the Brillouin-Zone integration. PAW potentials are applied to describe the electron-ion interaction with 10 valence electrons for Sr (4s^2^ 4p^6^ 5s^2^), 14 for Ru (4p^6^ 4d^7^ 5s^1^), 10 for Ba (5s^2^ 5p^6^ 6^2^), 10 for Ti (3p^6^ 3d^2^ 4s^2^), 6 for O (2s^2^ 2p^4^), 3 for Al (3s^2^ 3p^1^), and 9 for La (5p^6^ 5d^1^ 6s^2^). The in-plane lattice constant of each supercell is fixed as 3.871 Å^20^ to simulate epitaxial growth on a SrTiO_3_ substrate.

## Results and Discussions

**Figure 2:**
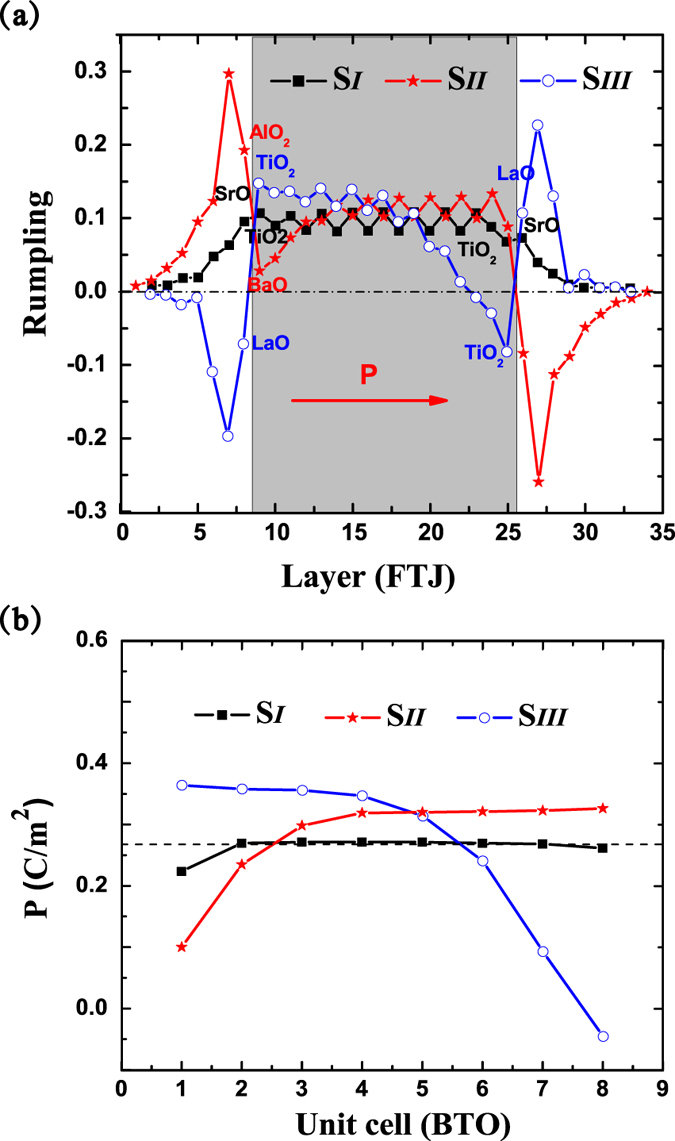
(**a**) Rumpling of cations with respect to oxygen atoms for relaxed FTJs with an initial positive ferroelectric distortion. (**b**) The profile of local polarizations within the barrier. Black squares, red stars, and blue circles correspond to structure S_*I*_, S_*II*_ and S_*III*_, respectively. The arrow in (**a**) represents the direction of polarization, the shadow region stands for the barrier, and the dashed line in (**b**) denotes the polarization of bulk BTO.

We start with an out of plane FE displacement obtained from the bulk BTO in the junction, then the out-of-plane lattice constant of each supercell is relaxed, together with the ionic relaxation. The relative cation-anion displacements within the electrode SRO and the barrier BTO are obtained based on the optimal structures and shown in Fig. 2(a), where the polarization of barrier points to the right. Squares correspond to the case of no atomic insertion, where the displacements are nearly symmetric with respect to the middle layer of the barrier, which is consistent with the result of ref. 20. As shown in Fig. 2a, without the inserted polar layer, screening of bound charges in such conventional FTJs arises mainly from the electronic screen from metallic electrode, and the rumpling in electrode SRO is always positive. However, when we insert (LaO)^+^ or (AlO_2_)^−^ within the interface, rumpling in the electrode changes significantly. For (LaO)^+^ in S_*III*_, the rumpling in the left electrode becomes negative. For (AlO_2_)^−^ in S_*II*_, the rumpling in the right electrode becomes negative. This grantees an effective screening of the inserted positive charged (LaO)^+^ or negatively charged (AlO_2_)^−^. The rumpling within the barrier is nearly symmetric with respect to middle layer in the conventional FTJ (in S_*I*_). When (LaO)^+^ is inserted, the rumpling in the barrier changes greatly, accompanying with the decrease of polarization and the appearance of domain wall. The local polarization of each unit cell in BTO is estimated using a model based on Born effective charge^30^ as follows, 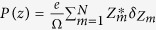, where N is the number of atoms in the primitive unit cell, 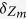 is the displacement of the m*th* atom away its position in the symmetric structure, and Ω the volume of unit cell. Although the method based on the Born effective charges calculated for bulk BTO cannot provide a quantitative accurate description of the local polarization in heterostructures. Nevertheless, it can give an estimation on the local polarization of FE barrier in tunneling junctions^31^. From Fig. 2(b), one can see that the polarization of the middle layer of the barrier is increased when the atomic monolayer is inserted, and the polarization of the middle region of the junction S_*I*_ approaches the bulk polarization of BTO. Due to the appearance of a FE domain wall near the right interface in structure S_*III*_, this leads to the decrease of the average polarization of the barrier. After a simple summation on the local polarizations, the average barrier’s polarizations are 0.26 C/m^2^, 0.28 C/m^2^ and 0.25 C/m^2^ for structures S_*I*_, S_*II*_ and S_*III*_, respectively. Therefore, the interfacial (AlO_2_)^−^ inserted-layer in structure S_*II*_ will raise the average polarization, while the (LaO)^+^ inserted-layer in structure S_*III*_ will lower the average polarization. To give a deeper understanding on the variation of the barrier’s polarization with the introduce of an atomic insertion at the interface, the electronic structure and charge transfer across the interface are given and discussed in latter paragraphs.

**Figure 3:**
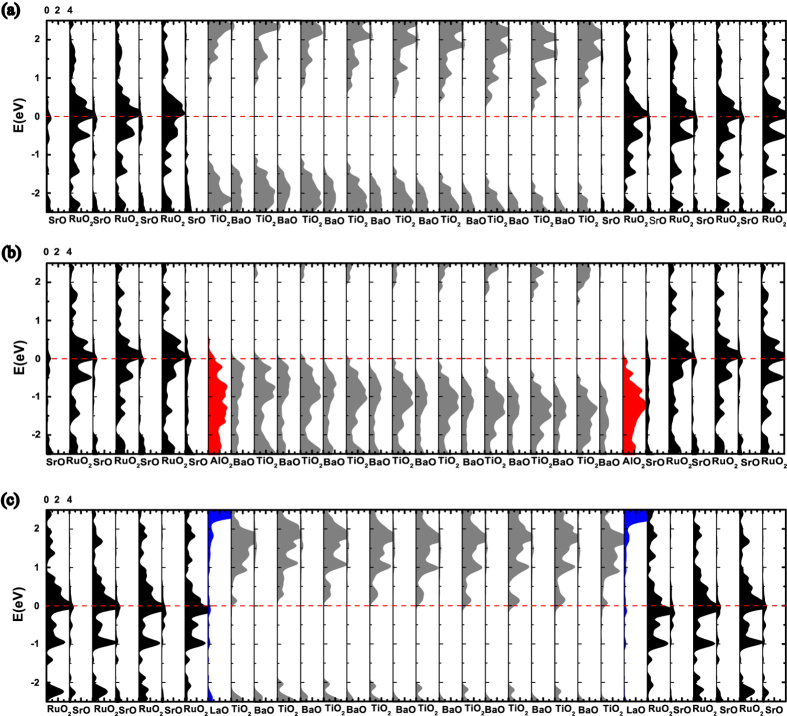
Layer-resolved DOS of FTJs (a) without insertion (structure S_*I*_), (b) with (AlO_2_)^−^ insertion (structure S_*II*_), and (c) with (LaO)^+^ insertion (structure S_*III*_). The horizontal red line denotes the Fermi energy.

**Figure 4:**
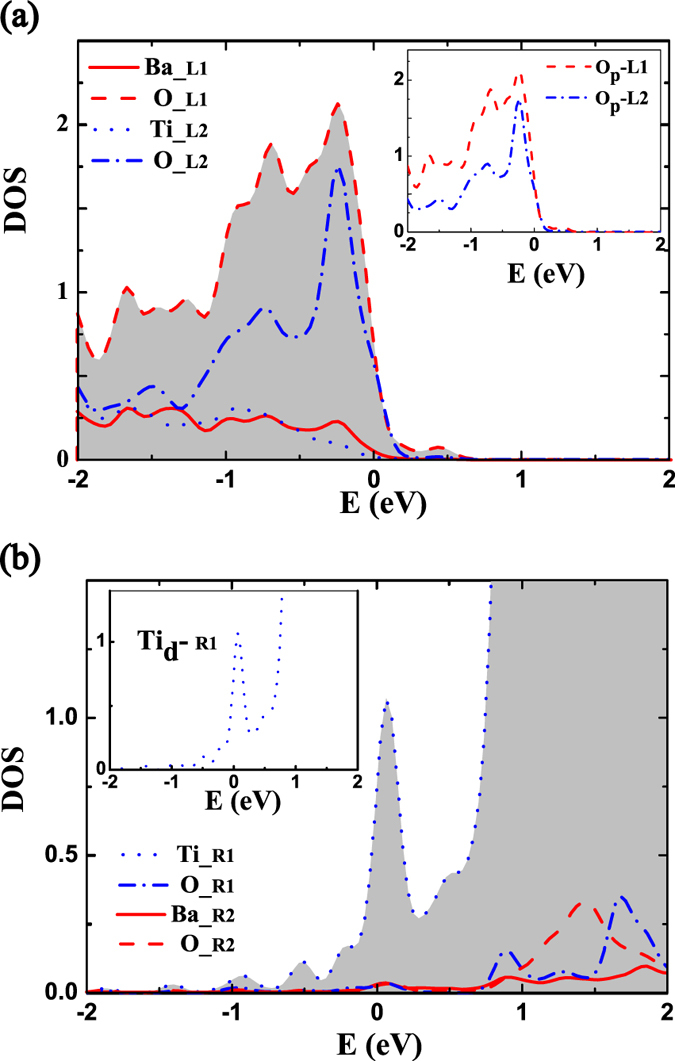
(**a**) PDOS of the left interfacial Ba, Ti, and O atoms in the optimized structure S_*II*_. (**b**) PDOS of the right interfacial Ba, Ti, and O atoms in the optimized structure S_*III*_. The inset in (**a**) shows the DOS projected onto O 2*p* orbitals at the left interface for Structure S_*II*_, and the inset in (**b**) shows DOS projected onto 3*d* state of Ti atom at the right interface for Structure S_*III*_. The subscript of atom symbol L1, L2, R1 and R2 denote the layer where the atom locates.

The layer-resolved density of states (LDOS) for FTJs with different interface configurations are plotted in Fig. 3. In FE films with either interface structures and for the polarization pointing to the right, the alignment of conduction-band minimum (CBM) and the valence-band maximum (VBM) across each layer of the barrier become curving. The bended alignment of CBM (or VBM) is triggered by the occurrence of depolarization field within the barrier, and the slope is proportional to the magnitude of internal depolarization field. Compared with the LDOS of S_*I*_ in Fig. 3(a), the Fermi level shifts to the top of valence band for the FTJ with (AlO_2_)^−^ interface insertion in Fig. 3(b), while in Fig. 3(c) for the case of (LaO)^+^ insertion, the Fermi level will shift to the bottom of conduction band. And the interfacial layer on the side of BTO becomes more conductive with respect to the case without atomic insertion, it indicates that there is a net charge transfer across the interface. As the (AlO_2_)^−^ monolayer is inserted in the interface, one can easily find that the electrons transfer from the barrier to electrode in Fig. 3(b), and this leads to the hole-doping at the interfacial layers of BTO. However, for the case of (LaO)^+^ insertion, the electron doping at the interfacial layers of BTO occurs, which results in the electrons shifting from electrode to the barrier. The projected density of states (PDOS) of the interfacial Ba, Ti, and O atoms are shown in Fig. 4. To see the occupation state of the transferred charges perceptibly, the projected DOS of left interfacial atoms and the right interfacial atoms within barrier BTO are shown for (AlO_2_)^−^ and (LaO)^+^ interfacial insertion, respectively. Bear in mind that the spontaneous polarization is chosen and fixed pointing to the right, unless otherwise specified. From Fig. 4(a), it is found that the holes mainly occupy at O 2*p* orbitals both at BaO layer and TiO_2_ layer. On the counterpart of (LaO)^+^ insertion, the transferred electrons almost site on Ti 3*d* orbitals, as shown in Fig. 4(b). Up to here, we obtain that the interfacial hole doping raises the barrier’s polarization, while the interfacial electron doping will suppress the polarization of barrier of FE heterostructures. However, it is well known thing will be different for the bulk BTO^32^, where the uniform carrier doping is always against the stability of ferroelectricity.

**Figure 5:**
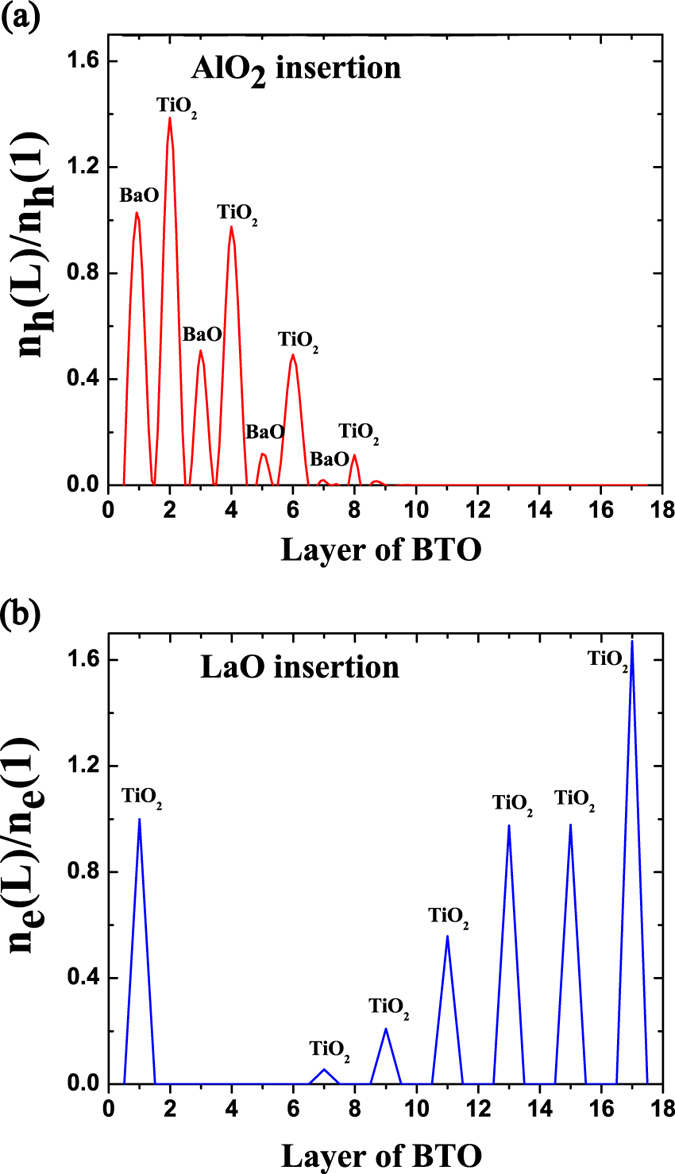
Distribution of carrier doping in the barrier for the FTJ (a) with (AlO_2_)^−^ insertion, and (b) with (LaO)^+^ insertion. Here, the amount of charge doping of each layer within the barrier is reduced based on that in the leftmost interfacial layer.

The distributions of transferred charges within the barriers for the case of interfacial (AlO_2_)^−^ insertion and (LaO)^+^ insertion are given in Fig. 5(a,b), respectively, where the number of charge doping in the leftmost layer is reduced to unit, and the amount of charge doping in other layer is a relative value with reference to that in the leftmost layer. As has also been illustrated in Fig. 4, the electrons are extracted from the barrier to electrode, and the holes are almost site on oxygen atoms for the case of (AlO_2_)^−^ insertion. From Fig. 5(a), one can see that the holes are asymmetrically distributed on the left side of barrier, which is resulted by the mediation of the spontaneous polarization. If the barrier stays at paraelectric state and with (AlO_2_)^−^ insertion, then the holes will symmetrically distributed at both the left and right side due to the mirror symmetry of the system. Under the action of an intrinsic electric field, the holes are easily to hop between O *p* orbitals, while the electron hopping between Ti *d* orbitals is difficult owing to the long distance between neighboring Ti atoms compared with the distance between neighboring O atoms along the direction of field. As reported in ref. 25, the hopping matrix elements between neighboring O *p* orbitals show no significant discontinuity. Then the theoretical high mobility of hole in the FTJ and electrons’ transferring to the electrode will help to screen the bound charges of the barrier, and further enhance the barrier’s polarization. In experiment, holes will easily be trapped in oxygen vacancies in the heterointerface which will reduce their mobility^33^. Therefore, the discrepancy between experiment and theory needs to be intensive studied by additional experiments and theories^25^. From Fig. 5(b), it is found that the electron inhabits the Ti d orbitals and there is no doping charge in the BaO layer. The depth of carrier penetrating into the barrier is large as compared with that in case of (AlO_2_)^−^ insertion in Fig. 5(a). Furthermore, the concentration of electron doping in Fig. 5(b) shows abnormality between the 2*nd* and 3*rd* TiO_2_ layer from the right, which is caused by the occurrence of FE domain wall within the barrier, and is consistent with the distribution of local polarization in Fig. 2(b). Consequently, the average barrier’s polarization will be suppressed in the FTJ with interfacial (LaO)^+^ insertion.

In summary, the atomic monolayer insertion at the interface of a typical SRO/BTO/SRO FTJ was investigated by the use of the first-principles calculations. The local polarizations are calculated based on the Born effective charge method, and it is found that the interfacial (AlO_2_)^−^ insertion is in favor of the enhancement of FE polarization. Through the analysis of electronic structures and the carrier doping within the barrier, the increase of barrier’s polarization for (AlO_2_)^−^ insertion is attributable to the hole doping near the interfaces, and the comparatively short penetrating length of the doped holes. Theoretically, the electron doping and hole doping have the opposite effect on the polarization of FE heterostructures. This is different from the doping effects in the bulk, where the carrier doping is always detrimental to the ferroelectricity. Therefore, the atomic monolayer insertion at the interface may be an effective way to enhance the polarization in FE heterostructures, and then improve the performance of FTJ-based nano-transducer. Further experimental studies should be carried out on the mechanism of the transportation of charges within the barrier, and a more practical FTJ with asymmetric electrodes or with asymmetric interfacial structures should be adopted for achieving a large TER.

## Additional Information

**How to cite this article**: Wang, P.-Z. *et al*. First-principles study of interface doping in ferroelectric junctions. *Sci. Rep.* **6**, 24209; doi: 10.1038/srep24209 (2016).
